# The Course and Diagnosis of Moon-blindness in Army Horses

**Published:** 1899-04

**Authors:** 

**Affiliations:** Marburg


THE COURSE AND DIAGNOSIS OF MOON-BLINDNESS IN ARMY HORSES. By Novotny, in Marburg. The following observations were made between August, 1896, and June, 1898, a period during which examination with the ophthalmoscope has been obligatory.

In the course of this period forty-four supply horses with eye-defects have been brought to the regiments. Among these there was one with a clouded condition of the vitreous body and cataract of the front capsule; three with clouded condition of the vitreous body without further change, and two with cataract centres without clouded lenses. All six were disqualified on account of moon-blindness. The four horses with clouded lenses showed no perceptible disease; the two with cataract centres a very slight atrophy in the eyeballs affected. Four of the horses were taken back by the dealer; two (one with cataract centre and one with clouded eyeball) were, for some unknown reason, retained. These latter have up till now shown no signs of inflammation perceptible to the naked eye. The central cataract of one has, however, increased, and cataract points have been plainly visible on the other for three months. In three other horses a similar cloudiness, without causing any visible inflammation, has developed in one and one-half to two years with a total gray cataract on the eye affected.

Eighteen other horses were affected in one eye with partial cataract of the front and three with partial cataract of the back capsule. In all the twenty-one cases, while no decay of the eyeball was observed, an active reaction of the pupils could be shown, and a most exact investigation of the background of the eye and the other parts gave a negative result. Six of these horses came from a supply-station in Hungary, and the question whether these horses had ever had traumatic, symptomatic, or periodic inflammation of the eye brought a negative answer. It is barely possible that the animals had had such a slight inflammation that it was overlooked. Of these twenty-one horses, four have been affected since with internal periodic inflammation in one eye and two in both eyes. In no case could this be detected with the naked eye. The test with the ophthalmoscope showed much cloudiness in the vitreous body and a very slow reaction of the enlarged pupils, but perfect transparency in the cornea and lens. In one case total blindness has followed as the result of a gray cataract on the right eye; the other horse shows no visible changes in his left eye.

In fifteen other horses under my observation up till now no inflammation can be detected. The cataract of the capsule has remained unchanged.

Seventeen horses showed pigment-specks, mostly on the front lens capsule. One of these horses which showed slight atrophy on the right eye, but active reaction of the pupil and normal appearance of the iris and background, has remained nevertheless sound in both eyes, and has shown as little inflammation or other changes as the other horses. Every four months the regiment horses are examined, and I have found individual cases of total or partial gray cataract which could be recognized with the naked eye. These animals had never had either traumatic or other inflammation of the eye. For this reason I began to examine all regimental horses oftener with the ophthalmoscope, and gained the following results:

In the course of four years six horses were found to be affected with cloudiness of the vitreous body, but in the other parts no other changes were detectable. Two of the same developed a total gray cataract in the course of one and one-half years. In one case a partial central cataract developed, which remained unchanged for thirteen months; two other horses became blind in the affected eye after having suffered several characteristic attacks of periodic inflammation. In one horse the cloudiness observed December 27, 1897, remained unchanged. No symptoms of inflammation have been detected in the affected eye. A horse which had a periodic attack of nerve inflammation (iritis, cyclitis, and chorioditis), causing contraction of the pupil, with synechia and later the formation of a gray cataract, showed in the left eye cloudiness of the vitreous body, which, up till now, has remained unchanged.

Early in 1895 the so-called "mouth-ache," which had been brought in with some Hungarian horses, appeared as a severe epidemic in Marburg. Some eighteen horses fell sick, showing pustules, with subsequent boils on the skin of the head and neck. In order to bring the epidemic to a speedy end all the healthy horses were injected with it. Of the thirty-two sick horses, two had conjunctivitis, with keratitis and iritis in one eye. The latter caused the collection of exudation in the front optic chamber. The greatly swollen conjunctiva showed in places papillomatous growths, whose degeneration occasioned the formation of flat, sharp-edged swellings, together with a strong, purulent secretion. At the close of these inflammatory symptoms both horses, in the eyes affected, showed a synechia, and, when this had been removed with atropin solution, a partial cataract of the front capsule. According to Dr. Knafletch, this trouble was probably a result of the mouth-ache; and this was confirmed by the fact that four days later several horses in the same lot were similarly affected. In these cases iritis occasioned synechia and a small cataract on the capsule. As the horses were removed, I had no opportunity to observe further developments.

Traumatic inflammation of the eye, with or without injury to the cornea, which, when no injury can be detected, are characterized by the gathering of exudation in the front chamber, leave such changes in the eye which has been affected that it is hard for the uninitiated to decide whether the same are to be attributed to symptomatic inflammation from which the animal has recovered. In such cases only decided atrophy of the eye, periodical internal inflammation, and, in the case of partial cataract only, the cloudiness of the vitreous body and changes in the retina can decide what kind of inflammation has caused the observed defects. Why the clouding of the vitreous body is sufficient to justify the diagnosis of moon-blindness is clear from the fact that symptomatic inflammation begins always on the outside, proceeds from front to rear, and occasions, first, trouble in the front uveal tract, to which in most cases it remains confined. Traumatic inflammation in ordinary cases does not affect the back uveal tract, or, if severe contusions have occasioned inflammation, the whole uveal tract is affected at the same time, and general changes in the eye are the result. On the other hand, internal periodic inflammation while arising can confine itself for a long time to the front or back uveal tract, and advance either from within toward without, or the reverse. Since atrophy of the eyeball is not perceptible after the first attack of internal inflammation, and a period of unchange, lasting eight to twelve months, usually occurs between the attacks in such cases where no striking changes in the eye are detectable after an attack of internal inflammation, or where they are confined to the front uveal tract, it is not possible to determine what kind of inflammation causes the partial cataract on the front capsule and, eventually, a partial synechia behind. As a result of this difference in the course of the disease, many defects, such as partial cataract on the front capsule, cannot be regarded as a sign of periodic inflammation from which the animal has recovered if the changes characterizing this trouble cannot be determined.

For the sake of completeness it ought to be added that two horses which were examined and showed no signs of disease under the ophthalmoscope afterward became blind—one in five, the second in nine months. I was not spared an unpleasant experience, because many dealers and breeders seem to think that when no defects are detected with the ophthalmoscope the eyes of the animals must necessarily remain sound during their lifetime.

The great number of military horses found with eye-defects during the last two years, together with a still greater number of cases of moon-blindness in horses which were accepted and showed no defects at the time of examination, shows the baselessness of the hope that obligatory examination of the eye with the ophthalmoscope can eradicate the trouble in army horses. It would, however, be unjust to make the examining veterinarian responsible for this or to charge him with ignorance in the use of the ophthalmoscope, since everyone knows how difficult it often is to examine the eye with this instrument, and a slight cloudiness on the periphery of the lens can easily be overlooked.

The method of investigation with the ophthalmoscope will certainly be improved in many points by general use, and certainly, in the opinion of many veterinarians, bring about a change in the diagnosis and view of moon-blindness which will influence the interpretation of the present legislation concerning the subject.—*Idem.*

